# The Development and Feasibility Study of a Multimodal ‘Talking Wall’ to Facilitate the Voice of Young People with Autism and Complex Needs: A Case Study in a Specialist Residential School

**DOI:** 10.1007/s10803-020-04476-6

**Published:** 2020-04-08

**Authors:** Norah Richards, Laura Crane

**Affiliations:** 1 Prior’s Court School, Thatcham, UK; 2 Centre for Research in Autism and Education (CRAE), UCL Institute of Education, University College London, 55-59 Gordon Square, London, WC1H 0NU UK; 3 City University of London, London, UK

**Keywords:** Autism, Complex needs, Intellectual disability, Communication, Pupil voice

## Abstract

Limited research has examined methods to investigate the views, preferences and experiences of young people with autism and complex needs. The aim of this study, based at a specialist residential school in England, was to develop and pilot an innovative method for this purpose—a ‘Talking Wall’—that was trialled over a 6-month period. Thematic analysis of data from focus groups and semi structured interviews with staff, combined with structured observations of pupils, resulted in three key themes: supporting the expression and evaluation of emotions that underlie preferences; recognising the impact of transitions; and the important role of familiar adults in interpreting communication bids. These positive, initial findings suggest the Talking Wall approach merits further development and evaluation.

**Electronic supplementary material:**

The online version of this article (10.1007/s10803-020-04476-6) contains supplementary material, which is available to authorized users.

The United Nations Convention on the Rights of the Child (1989) states that all children have the right to be heard and to have what they say taken seriously. However, protecting and enhancing these rights when the child has autism along with communication, social, sensory, mental, emotional and/or physical needs (hereafter referred to as ‘complex needs’) undoubtedly presents challenges. Professionals have a legal obligation to develop and use appropriate means to elicit the views of children and young people, regardless of their perceived ability (see Special Educational Needs and Disability Regulations 2014 and Children’s Act 2004). Indeed, a report on the wellbeing and rights of children and young people educated in residential special schools in England (Pellicano et al. 2014) concluded that it is vital to understand young people’s views around day to day matters, which are as meaningful and relevant to those young people as decisions about schooling; even though the latter, arguably, have more profound consequences for their future independence.

Generally, when we want to know what other people think, the most reliable information comes from what they say or do. This may include behaviours such as facial expressions, body language, gestures and verbal or auditory cues (Hall and Knapp 2013). However, this kind of information is limited if it cannot be effectively or reliably understood, which is often the case for young people with autism and complex needs. Sometimes it is necessary to ask others who know them well to provide their interpretation of the information they provide (Greathead et al. 2016). This raises the question of veracity: how can we know what a person wants if nobody confidently speaks their language?

There has been limited research considering effective methods for eliciting pupil voice in young people with autism. Recent reviews by Nicholas et al. (2019) and Tesfaye et al. (2019) synthesised methods used to obtain the perspectives of young people with disabilities, whilst considering the relevance and applicability of these for young people with autism. Although a good starting point, both reviews noted that most pre-existing studies evaluated methods for participants with verbal skills, predominantly using semi-structured interviews (Nicholas et al. 2019; Tesfaye et al. 2019). Whilst the use of augmentative and alternative communication systems (AACs) such as Picture Exchange Communication System (PECS), sign language, or speech generating devices (SGD) may go some way to addressing such barriers to participation, a certain mastery of these tools is required, thereby still precluding those young people who have not reached this ‘level’.

Aspects of these reviews that can be applied to pupils with autism and complex needs include an emphasis on collaboration with key stakeholders who are familiar with the young people, to create optimal environments for self-expression and potentially more reliable interpretation (Tesfaye et al. 2019). Further, the use of a flexible approach to research that evolves through collaboration, rather than following a prescriptive protocol, was emphasised (Tesfaye et al. 2019). Nicholas et al. (2019) discussed specific methods that may be useful for young people with autism and complex needs including Sensecam, Timeslips, Photovoice and ‘Talking Mats’. Although Sensecam (a wearable camera) and Timeslips (pictures used to create narratives) are arguably not effective methods for young people with autism and complex needs, Nicholas et al. (2019) suggested that they do offer insight into the benefits of using graphic based methods. Photovoice is one such method, which involves young people taking photographs to respond to the question: “what is important to me?” Ha and Whittaker (2016) evidenced some success using Photovoice with children with autism, but concluded that due to the ambiguous nature of the photographs, analysis of the contents alone was insufficient and a multi modal approach was required (e.g., supplementing photographs with interview data). Cluley (2016) adapted this method to include photographs directed by participants with profound and multiple learning disabilities, with photographs taken by carers who acted as co-researchers. This approach could offer some possibility of success for participants with autism and complex needs, due to its inherent flexibility and limited resource requirement.

Nicholas et al. (2019) noted that another visual method, ‘Talking Mats’ (involving the sorting of visual cards into ‘likes’, ‘dislikes’ and ‘in-between’) has promise for pupils with autism and complex needs. Success using this approach with people who have complex needs has been evidenced (Bradshaw et al. 2018; Gridley et al. 2014; Hallberg et al. 2013), and it has been used with children with autism (albeit with mixed success: see Cummins et al. 2018). Potential limitations of this include the pre-requisite understanding of ‘like and dislike’ and difficulties considering events that are in the past or out of context. Furthermore, the limited inventory of pre-prepared visuals given to each young person could predefine the parameters of the conversation (Doak 2019). In the specific context of autism and complex needs, ‘Talking Mats’ used as a sole measure risks being a tokenistic approach.

The use of multiple measures in combination has been shown to have promise in preverbal typically developing children. The Mosaic Approach (Clark 2010; Lundy 2018) echoed the approaches discussed above by gathering evidence from both the child (drawings, photographs, interviews) and their familiar adults (observation notes and photographs). Clark (2010) stated that although this approach focused on typically developing children, it was flexible and could be individualised, so there was scope to extend it to young people with intellectual disabilities.

In most cases in which pupil voice has been elicited, the communication methods used in the research were already known and practiced by the participants (Tesfaye et al. 2019). Hill et al. (2016) evidenced some success using three novel methods (school preference cards, a diamond ranking activity and an adapted graffiti wall) with young people with autism and intellectual disabilities (see Pellicano et al. 2014). The school preference cards comprised photos of the young people’s everyday environment, which they sorted into ‘like’, ‘dislike’ and ‘don’t mind’. If strong reactions were noted, participants were also asked to match an emotion card. The researchers noted that, by using familiar images, the photographs captured participants’ interest, created a bond between the researcher and participant, focussed attention, and communicated concepts. Hill et al. (2016) further noted that some of the cards acted as emotional triggers, for example a picture of the entrance to the boarding house elicited a ‘sad’ response from one young person, who went on to self-report that he missed his parents. Limitations to this method included the fact that the photographs needed to communicate the desired concept clearly, and that a degree of interpretation was required on the part of the researcher.

Hill et al.’s (2016) diamond ranking activity allowed participants to discuss and ‘rank’ statements about areas of school life without being directly interviewed, something that was recognised as particularly positive for young people with autism who did not like direct or uncomfortable questions. However, a certain level of verbal reasoning was required to complete this activity and, as such, it may not be an effective method for those young people with complex communication needs. The final method trialled by Hill et al. (2016) was an adapted Graffiti Wall. Here, the young people were given post-it notes of two different colours. They were asked to write or draw all the good things about school on one colour and all the bad things, or things they did not like, on a separate colour (note: for those children who could not write, symbols and communication aids were used to enable key workers to scribe for them). The post-it notes were stuck on a wall and shared with a group to support wider discussion. Here, Hill et al. (2016) noted the principal limitation, in that those young people requiring assistance may have inhibited negative views, or indeed had those views inhibited for them due to the provision of a restricted set of symbols.

Each of the methods trialled by Hill et al. (2016) extended the concepts behind the ‘Talking Mats’ approach, in that they were visual and allowed for the independent or supported categorisation of events, activities and concrete objects into the categories of ‘like’, ‘neutral’ and ‘dislike’. It is also noteworthy that Hill et al. (2016) used a ‘framework’ approach by using an observation method based on a novel adaptation of the criterion referenced SCERTS framework (Social Communication, Emotional Regulation and Transactional Support; Prizant et al. 2006). This approach was supplemented by unprompted ethnographic observation notes to provide a rich narrative (see also Doak 2019; Lyons et al. 2015). Hill et al. (2016) commented that this framework helped to identify patterns of communication for those children with the highest needs, concluding that some communication partners were better at facilitating the young person’s active participation. In most cases, this communication partner was someone very familiar with the young person.

Despite reviewing a number of suggested methods for eliciting the voices of young people with disabilities, both Tesfaye et al. (2019) and Nicholas et al. (2019) concluded that there is not enough research addressing this topic (see also Russell et al. 2019). In autism education, one way to address this issue has been proposed by Parsons et al. (2013). They promoted a model of collaborative partnership between research and special schools, arguing that joint working—between researchers and teachers who know the pupils and school context well—will address the research-practice gap.

The aim of this current, preliminary case study was to extend existing research by developing and piloting an innovative method for eliciting the voices of young people with autism and complex needs in a specialist residential school in the South of England. This project was undertaken in response to this issue being identified as a priority for action within the school, and the research was conducted by a practitioner–researcher (NR) alongside an independent academic researcher (LC). Two research questions were investigated: (1) How can the use of a shared multimodal physical space be used to promote and support the elicitation of the views, representing both positive and negative experiences of young people with autism and complex needs in a specialist residential school? (2) How can the school use the evidence generated from this to listen, respond and ensure the protection of the rights of the young people?

## Method

### Context

A case study was conducted at a specialist residential school in the South of England, Prior’s Court School. Prior’s Court caters for young people between the ages of 5–25 years (hereafter referred to as ‘young people’). All of the young people attending the school have formal diagnoses of autism and learning disabilities. They also all have complex needs; most students are minimally verbal, and many have associated diagnoses or exhibit behaviours that challenge.

The school follows a ‘waking day’ curriculum, supporting the young person from the moment they wake up until they go to bed, operating across both residential care and education settings. The school applies a consistent learning approach (developed internally), using an evidence-based toolkit of skills that support the young people to make sense of the world around them, and promote communication and independence. All young people have access to an individualised communication system ranging from objects of reference, which are objects that are systematically used to represent people, places, objects and activities; True Object Based Icons (TOBIs), which are life sized photographic representations of objects; Picture Exchange Communication System (PECS); Speech Generating Devices; and Signalong, which is a key-word signing system based on British Sign Language used by all young people. Although some element of choice is implicit at appropriate times within the ‘waking day’ curriculum (e.g., choosing clothes, food, certain activities), the school was particularly interested to develop more explicit methods for young people to make their preferences and views known about a wider range of topics, regardless of their preferred method of communication and the ‘level’ to which they accessed that method. The school tasked the researcher (NR), a qualified teacher working as an autism education practitioner in the Speech and Language Team, to lead a case study to facilitate this process. The second author (LC) is an independent academic researcher.

From the literature review presented above, no single method for eliciting views, experiences and preferences emerged as effective for all pupils, although this was to be expected given the heterogeneity of this cohort. Therefore a ‘grounded’ evolving approach was taken, combining various elements of previously trialled approaches (e.g., Photovoice, ‘Talking Mats’, School Preference Cards, adapted Graffiti Wall, Mosaic Approach) into a multimodal approach. In discussion with the school, the proposal was to pilot adapted graffiti wall spaces as ‘interactive collection points’ for text, artefact, image, photographic or audio based evidence of the young people’s views (hereafter referred to as ‘Talking Walls’). To review the efficacy of the method, a detailed observation schedule was created, drawing upon the structure provided by the SCERTS framework (Prizant et al. 2006) and supplemented by ethnographic field notes (cf. Hill et al. 2016).

### Participants

#### Young People

Ten young people participated, all of whom had autism and complex needs. Referring to informal and non-standardised assessment data, it was determined in agreement with the Speech and Language Therapy Department that 7 of the participants met the working criteria of minimally verbal, which Tager-Flusberg and Kasari (2013) distinguished from preverbal as having little or no functional expressive speech over the age of 5, using perhaps fewer than around 30 words. Although the remaining three participants did not meet the criteria to be defined as minimally verbal, they each presented difficulties with communication that compromised their ability to make their preferences known. Six of the young people were male and 4 were female, and their average age was 19 years (ranging from 15–26 years). Many also had additional diagnoses, including epilepsy (n = 4), attention deficit hyperactivity disorder (ADHD, n = 2), Tourette’s syndrome (n = 2) and additional medical needs (n = 1).

#### Staff Members

To include as many stakeholder voices as possible, a broad range of school staff who work most closely with the young people were invited to join the study. Eighteen staff members accepted: 7 of whom worked in an educational capacity, 10 of whom worked in a residential capacity and 1 therapist who worked across both settings. Eleven of the staff members were women, and seven were men. The sample comprised 4 teachers, 2 residential home managers, 1 speech and language therapist (SLT), 1 SLT assistant and 10 autism practitioners, all of whom support the young people directly. Of the original 18 staff, 6 went on to participate directly in piloting the Talking Walls in 4 areas. (Note: pseudonyms have been used throughout, to protect the identities of all participants.)

### Materials and Procedure

The study followed a three-phase approach: Phase One comprised focus groups and interviews with staff; Phase Two comprised the development of the Talking Walls; and Phase Three comprised an evaluation of the Talking Walls, involving interviews with staff alongside observations of the young people.

#### Phase One: Design

Four in-person focus groups (range 3–5 participants) were conducted with staff members to determine the working format of the proposed Talking Wall method. A focus group guide was created for the study, following a semi-structured format. First, examples of the adapted Graffiti Wall (Hill et al. 2016), the Mosaic Approach (Clark 2010) and Photovoice (Cluley 2016) were presented to the group, to stimulate discussion. Then, discussions centred around four key topics: (1) what the Talking Walls should look like; (2) where the spaces should be set up; (3) when/how the use of the spaces should be encouraged; and (4) what should happen once the young people have expressed their views. A number of related prompt questions were used to probe for further information, if and when necessary. The focus groups lasted an average of 44 min (range 40–45 min). To accommodate shift workers, two semi-structured, one-to-one interviews (following the format of the focus group) were also carried out. These lasted, on average, 49 min (range 42–55 min). All discussions were audio recorded and transcribed verbatim.

Data from the focus groups and interviews were analysed together, by the first author, using thematic analysis (Braun and Clarke 2006). An inductive approach was used, meaning that data were not coded into a pre-existing coding frame. Data were analysed at a semantic level, with themes identified from the explicit meaning of the data. Analysis involved: transcription; actively reading and rereading the transcripts; generating initial notes; manually coding the data set; sorting the codes and identifying potential themes and subthemes. Themes were refined using an iterative process: the second author independently analysed the data and the authors discussed possible themes to ensure reliability.

#### Phase Two: Development

Four pilot areas were selected by the school: two in classrooms and two in corresponding residential houses.

Following the focus group discussion and initial interviews, materials for the Talking Walls were produced. This included a social story (Gray 2010) to show how to use the Talking Wall, emotion symbols for young people, and guidance notes for staff. The guidance notes gave a brief overview of how to use the Talking Wall, focusing primarily on the need for supporting adults to model emotional language and the importance of labelling a young person’s emotion as they appeared to be experiencing it. Notes were presented during staff training sessions, scenarios were modelled, and staff were given the opportunity to ask questions (see Supplementary Material for full details). The guidance notes were also pinned to the Talking Wall for reference and these recommended that items pinned to the Talking Wall should be transferred into a scrapbook on a regular basis. Staff were encouraged to use the wall whenever possible.

#### Phase Three: Evaluation

The evaluation phase comprised structured observations of the young people plus semi structured evaluation interviews with staff. A total of 21 observations of young people were carried out over a period of 2 weeks, lasting on average 35 min (range 15–55 min). The practitioner–researcher worked together with the SLT team to complete the SCERTS framework (Prizant et al. 2006) ‘Worksheet for Determining Communication Stage’ for each Young Person. Complete consensus was reached. Participants were assessed as either ‘social partner’ (non-verbal or minimally verbal) or ‘language partner’ (more verbal) (Prizant et al. 2006). Observations were carried out using structured checklists modified from the social and language partner SCERTS schedules (Prizant et al. 2006). For this, the SCERTS Expression of Intentions and Emotions Worksheet (Prizant et al. 2006) was adapted to record the expressive strategies used by the young person from a list of defined socio-communicative behaviours (e.g., Requests desired object, Takes turns, Comments on object, Expresses emotion). Whether the young person used pre-symbolic means (e.g., eye-gaze, facial expressions, reaching, showing, or waving) or symbolic means (e.g., delayed echolalia, sign language, or a picture system) was recorded. To ensure the reliability of the coding, four observations were concurrently made by two speech therapists, representing 16% (104 min) of the total observation time. Discussions immediately following the observation period allowed for any differences in observation to be agreed. Only small differences were noted, for example an additional eye glance or vocalization—no material differences were noted. Ethnographic reflections supplemented the structured checklists (cf. Hill et al. 2016). As an employee at the school, the practitioner–researcher was able to spend considerable time across both residential and education settings, not only making informal observational field notes about the young people, but also getting to know them, in order to be accepted as ‘a familiar adult’. It was hoped that this would result in the young person behaving naturally when they were in attendance.

Out of the initial 18 members of the wider staff who took part in the Phase One focus groups, 6 directly piloted the Talking Walls. Four of these staff members were then interviewed via semi-structured interviews for an average of 16 min (range 6 min 22 s to 21 min). (Note: two staff members declined to be interviewed.) Discussions were based on the two broad questions of what had worked/not worked, and themes from the focus groups in Phase One were revisited. Recordings were transcribed verbatim and thematic analysis was used, following the same approach used in Phase One.

#### Procedure for Including the Young People in Research

As in previous studies (Hill et al. 2016; Loyd 2013; Preece and Jordan 2010), consent was conceptualised as a continuous process rather than a one-off agreement. Following parental written consent being obtained, the young people participating were made aware of a social story that included a simplified explanation of the project. Immediately before any observation, young people were given a verbal briefing and consent was sought verbally (where possible). Additional communication aids were used where necessary, including sign, symbols or any other preferred communication system. Key to monitoring consent was recognising that the young people may experience anxiety or discomfort when asked to express emotions or views, particularly if that would elicit a negative or uncomfortable feeling. Information sheets, direct teaching about how to use the Talking Wall, and social stories about expressing negative emotions aimed to facilitate the learning that it is ‘ok’ to say ‘I don’t like’. In all cases, observations clearly reflected if a young person displayed signs of discomfort and chose not to display their viewpoint (positive, negative or neutral). It was recognised that there was also a right ‘not to express an opinion’. A red ‘stop’ card was provided, which young people could touch at any time (as a sign that they did not wish to continue). Familiar adults and the researcher also monitored behaviour for any signs of discomfort and observations were terminated immediately upon signs of distress. Ethical approval for the project was granted via the Department of Psychology and Human Development at UCL Institute of Education.

## Results

### Phase One: Design

Five themes were identified during the focus group discussions undertaken in Phase One: (1) a personalised whole group approach is key; (2) a structured environment potentially restricts free expression; (3) questions regarding the young people’s ability to respond to the Talking Wall; (4) the multiple roles of staff; and (5) the physical management of the space.

#### Theme 1: A Personalised Whole Group Approach is Key

Staff were generally less concerned that the approach was evidence-based and more concerned that the Talking Wall allowed for the individual expression of each young person, no matter what their level of need. They felt that different approaches needed to be interwoven and questioned if this may compromise the fidelity of a ‘shared’ Talking Wall approach. Yet, they concluded that this was a necessary level of complexity.

I think we will just have to take the risk, because if we do it as individualised and they just have their own scrapbook and they take their own stuff and stick it or whatever, then they are not sharing with anyone. Nobody sees it. Unless you go and show people. But the board is something everyone can see.

#### Theme 2: A Structured Environment Potentially Restricts Free Expression

Staff felt that the highly structured environment (common in specialist schools) meant that the young people were predominantly given closed as opposed to open-ended choices, to reduce their anxiety. All staff agreed that the reduction of anxiety was paramount but that this potentially inhibited the young people’s views, mainly because the closed choice options were predetermined by staff. Staff agreed, however, that the choice options were selected by staff who were familiar with the young people and believe that they were acting in the young people’s best interests. Most staff felt that an additional method to elicit pupil voice would be a useful tool.

A lot of the time with schedules, we are very much pointing them in a particular direction, which we think they should be going in. Yes it is a good thing to do that. However, I think they should be given choice within that direction as well.

So, Luke was also given the choice – did he want a birthday party here or did he want to go out for a meal? Luke chose to have his party here. And I said that is a nice thing to do because you can include everybody within the house.

Staff also commented that a direct result of the TEACCH^1^ approach used within the school is that young people are taught to ‘match’ in order to follow a visual schedule. This may mean that any visual representations potentially become matching or sorting activities, which may inhibit the young person’s true expression.

#### Theme 3: Questions Regarding the Young People’s Ability to Respond to the Talking Wall

Staff talked extensively about individual young people and the responses they might expect. The most discussed topic was around the young people’s understanding of emotion. Staff differed in their interpretation of what the young people may or may not understand, may or may not physically experience, and whether or not the young people could differentiate between ‘like’ and ‘dislike’ (for the latter, most staff felt that they could not). This prompted further debate about the fact that positive and negative emotions underlie ‘like’ and ‘dislike’ and that in order to reliably elicit preferences we need to first work on emotions. This was analysed further into recognising physical feelings at the point of experience and labelling these physical feelings as emotion.

Additionally, staff felt that learned behaviours or the general bias around social desirability could override the young person’s true expression: staff expected most young people to select ‘like’ over ‘dislike’ because they tend to get a more positive response to ‘like’.

I always remember, when I first started working here, speech and language had just introduced Talking Mats… and they were doing a like/dislike with a young man, and he said he liked everything. So, they gave him a spoonful of English mustard on a tiny teaspoon and I was filming, and he still went “uurrgghh - like!”

Staff expressed a desire for additional training around the teaching of emotion. Staff also debated whether or not young people can recognise themselves in photographs, which led to questions around young people’s level of engagement. Some staff members reasoned that if there was no personal recognition, there was no connection and the Talking Wall would simply be a display board, managed by the staff and therefore tokenistic in nature. Other staff members held strong beliefs that the young people do recognise themselves in print and also enjoy and engage with their own photographs. They discussed examples of their young people actively commenting on their own photographs.

A further issue raised was that many young people have working memory or auditory processing difficulties and as such may simply select the first or last choice they were offered, making the approach tokenistic at best.

**Staff member**: so, if you gave them two choices, the last one you offered them would be the one they picked, if you then asked them again, the other way around, they’d still pick the last one—so make a different choice. Because that is the last one that they remember.

**Researcher**: Why do you think that is?

**Staff member**: It's sort of because that is how they have learned to do it, but also because if they pick the thumbs up, they get the right response from you… it’s quite frustrating, and sometimes it gets worse because I’ll go – ‘right, you’ve got 3 choices’ and I’ll be pointing, and they go with the one I’m pointing at. So, I have to do it without trying to do anything and it can be very tricky.

Staff were very clear that any expression had to be immediate, as the activity was happening. Waiting to discuss later would be out of context, meaningless to the young people and therefore unreliable and inaccurate.

So… if you went bowling, and you got the score card, at what point would you put it on your Wall? Because by the time you have come back here and you went to McDonald’s on the way home, it could be an hour or so later, by the time you have come back, that score card might not mean anything an hour down the line.

#### Theme 4: Staff have Multiple Roles

Staff discussed at length their roles in the process. These included: teacher (teaching a range of emotions); facilitator (supporting the young people to express themselves); collator (collecting and ensuring all forms of expression are recorded); caretaker (managing and preserving or protecting the Wall spaces on the young people’s behalf); and interpreter (knowing the young people well enough to read and understand the behaviours, facial expressions and body language and to label those responses with an emotion, yet at the same time, not making assumptions; as illustrated below).

… if you are at the zoo, you think it’s a great day out, the sun is shining and oh look! They are laughing at the penguins… Actually they are really anxious that there is a penguin just roaming around, or there is a bird, or we are in the butterfly house and the butterflies come and land on you. Oh! Yes, this is great sensory, oh! They are laughing, oh! They are happy… mmm, we don’t actually know that.

#### Theme 5: Physical Management of the Space

Discussion centred around how the Talking Walls may look. It was agreed that the basic Talking Wall should have three main areas: space for ‘like’, ‘dislike’ and ‘O.K.’. Staff agreed that as many types of media as possible should be used, including photographs, post-it notes, drawings, tickets from events, and maps/flyers from places visited. Staff concluded that the boards would, could and should look different depending on their setting (educational or residential) and agreed that the linking factor to create consistency is the use of a shared ‘scrapbook’ containing all of the postings across the educational and residential environments.^2^ All staff interviewed felt that the young people need to identify themselves via photographs on the board; nobody commented or felt that the boards should be anonymised. One of the main difficulties highlighted was how to represent ‘negative’ emotions or ‘dislike’, as staff felt that reminding young people of unpleasant events could heighten anxiety. It was suggested that an opaque pocket could be used in which to ‘post’ negative feelings:

I think with Alice, we could have somewhere where she sticks the ones she likes, and then something like an envelope where she can post the ones she doesn’t like—so that she doesn’t see them…

### Phase Two: Development

Phase Two comprised the creation by staff of a ‘Talking Wall’ in each of the four pilot locations (educational and residential). Four training sessions of 30 min were run during weekly staff meetings, where the materials for the Talking Wall were presented by the first author and a SLT. For the purposes of this study, it was agreed with the SLT team that the support given to staff surrounding the teaching of emotion would include both the modelling of emotion in oneself and the labelling of emotion in the young person. As a starting point, the six ‘basic’ emotions of happiness, sadness, fear, anger, surprised and disgust (Ekman 1999) were considered and it was agreed that, for the purposes of facilitating the Talking Wall, it would be too broad to focus on six emotions. Therefore ‘happy’ and ‘sad’ were selected, along with ‘worried’, which was considered the word most commonly used amongst staff to cover ‘fear’ and to some extent, ‘surprise’. In addition, the term ‘O.K.’ was selected to allow for the expression of a neutral feeling.

Although staff were animated during focus group discussions around the suggested format, a number of weeks passed before the Talking Walls were ready for operation. This could have been due to the fact that staff were less confident around setting up the areas, but more likely it was due to the fact that staff were operating in an intense, busy and challenging environment and that other physical, health or safety issues which arose took precedence (Parsons et al. 2013).

### Phase Three: Evaluation

First, the evaluation phase consisted of observations of the young people. Second, staff were interviewed.

#### Observations

Overall, the first author spent a total of 11 h undertaking 21 separate structured observations of everyday activities, both in educational and residential settings (average 30 min each, range 15 to 45 min). During observations, there were six successful uses of the Talking Wall; seven ‘missed’ opportunities and seven occasions when it would not have been appropriate to use the Talking Wall (e.g., when a young person struggled to transition back to the Talking Wall or when the researcher or familiar adult felt that the young person was showing signs that they no longer wished to be observed and requested a ‘break’). Out of the ten young people who took part in the Talking Wall pilots, two were not directly observed by the researcher during structured observation sessions. As previously noted, participants were assessed as either a social partner (n = 7) or a language partner (n = 3). This translated into 8 separate observations of social partner interaction and 13 separate observations of language partner interaction.

Table 1 summarises observational data and highlights patterns of communicative intent. In summary, these data demonstrate that *all* participants relied to an extent on the pre-symbolic means of proximity, eye gaze and facial expressions (comprising 34% of the observed bids for communication). These were always directed towards their familiar adult. Physical gestures such as reaching and pushing away also featured, most often when the observations were around concrete activities such as craft or food. Gestures (such as ‘showing’) appeared at the language partner stage, suggesting some evidence of joint attention. Incidences of echolalia were observed as expected in both groups, along with single word exclamations. Creative word combinations differentiated the social and language partner stages.

**Table 1:** Patterns of communicative intent

Communicative intent	Social partner		Language partner		Total occurrence	
	Number of occurrences	% of SP occurrences	Number of occurrences	% of LP occurrences	Total number of occurrences	% of total occurrences
Pre-symbolic means						
Shifting eye gaze	31	19	31	9	62	13
Facial expressions	21	13	36	11	57	12
Proximity	25	15	20	6	45	9
Simple motor action	17	10	21	6	38	8
Reaching	15	9	17	5	32	7
Pushing away	5	3	10	3	15	3
Re-enactments	5	3	10	3	15	3
Showing	1	1	12	4	13	3
Differentiated vocalisations	7	4	6	2	13	3
Variety of consonants and vowels	9	5.5	2	< 1	11	2
Pointing	2	1	7	2	9	2
Head shake	0	0	7	2	7	1
Giving	3	2	3	1	6	1
Self-injury	0	0	3	1	3	1
Waving	0	0	2	1	2	< 1
Crying/whining	0	0	1	< 1	1	< 1
Tantrum	0	0	0	0	0	0
Aggression	0	0	0	0	0	0
Head nod	0	0	0	0	0	0
Symbolic means						
Creative word combinations	0	0	65	20	65	13
Single words (spoken)	8	5	41	13	49	10
Immediate echolalia	6	4	20	6	26	5
Delayed echolalia	9	5.5	12	4	21	4
Total occurrence	164	100	326	100	490	100

To compliment the structured observations, unprompted ethnographic field notes were also taken by the researcher (Greathead et al. 2016; Nind et al. 2010). This process highlighted four additional key observations. Firstly, there was no spontaneous independent use of the wall, neither directly observed, nor reported. This is, arguably, not surprising as the Talking Wall practice needed to become a familiar routine before any independent postings would be expected. Yet several young people did show interest in the spaces; pausing and looking. Second, due to the potential power imbalance between the young people and their familiar adults, some young people may think that there is a single ‘right’ response in any given situation. During observations, several supporting adults commented that the Young Person was reporting that they ‘liked’ an activity in order to get a positive response. Third, all observed incidences of using the wall were generated after positive experiences. There was only one reported incidence of a young person writing ‘worried’ on a post-it, which they posted into the ‘dislike’ envelope. During observations, staff commented that it was easier to focus on positive language and they felt that paying attention to negative events heightens anxiety and potentially triggers behaviours that challenge. A final observation was that this research was initially aimed at autism practitioners who work one to one (and therefore most intensively) with the young people. However, it was noted that staff sometimes fluctuate, and not all supporting adults are ‘familiar’.

#### Interviews with Staff

Following the observation period, semi-structured interviews with four out of the six staff members who were actively involved in piloting the Talking Walls were carried out to evaluate the project from the staff perspective. Discussions centred around the two broad questions of what worked well and what did not work. In addition, themes were revisited from the focus groups in Phase One. Three main themes were identified from these data: (1) the expression of emotion; (2) the impact of transitions; and (3) the role of the supporting adult.

#### Theme 1: The Expression of Emotions

A concern that was consistently raised at each stage of the research was that the young people struggle to identify and express emotion. Staff acknowledged that although positive emotions were more regularly labelled, the Talking Wall has highlighted the importance of labelling negative emotions:

I’m more mindful of it now. When I’m working with certain young people, Luke for example, and he tells me that he is scared or he is worried, I say “Thank you for telling me you are scared!” Being positive about the negative. You know – its brilliant that you’ve told me, it’s not brilliant that you feel that, but we can work on it.

Another staff member noted the importance of emphasising that it is good to feel ‘o.k.’.

The other thing we tried to do is get some more mundane stuff, so the other day, Oscar went round and took photos of everyone and he took photos of Connor doing laundry, they printed it out right there and then we asked Connor how he felt and he said that he doesn’t care about laundry. So that was really good.

This example also highlighted the possibility of the Talking Wall creating the environment for a shared experience. The staff participant went on to explain:

So then it becomes an activity that the young people are doing to support the other young people so that staff don’t feel ‘oh I'm the one that has to take the photo’ and ‘I'm the one that has to do it’ – if it’s something that the young person is doing and they are enjoying it.

#### Theme 2: The Impact of Transitions

The Talking Walls were fixed in the educational and residential ‘houses’ and any events that occurred outside of these areas required the young person to transition back to the Talking Wall. Issues around timing were noted and staff discussed the importance of instant recognition/response (due to the potential effects of transitions or memory). To mitigate this effect, the use of an instant camera was trialled. Staff reported that this evidenced some success. For example, during a cookery class, one young person, who does not speak, appeared to enjoy making flapjack. She showed interest in an instant photograph of her eating the flapjack, which developed to show her own image in front of her whilst she was still eating. She then carried the photograph back to the classroom and her familiar adult, with a gestural prompt, supported her to post the picture onto the ‘like’ side of the Talking Wall. Her willingness to stand at the Talking Wall, glances towards her photograph and eye contact with her familiar adult were interpreted as evidence that she had seemed to enjoy the activity.

However, on several occasions, staff recounted that an event that would have been meaningful to ‘post’ on the Talking Wall was lost because there was no immediate return. For example, staff described how a trampolining activity that appeared to be ‘fun’ or ‘exciting’ had occurred just before lunch; by the time the lunch break finished, the young person had experienced a difficult situation and the positive emotions of two hours earlier were ‘lost’. Staff suggested that a more ‘portable’ approach might work, possibly using an electronic device for photographs and comments, but also noted that this would lose the concrete act of holding the photograph or artefact and posting it on the Talking Wall.

Staff also reported that the issues around transition were complicated by the impact of anxiety and described how a young person was engaged during a music therapy session and was able to self-report that she was “happy singing”. An instant photograph of her at the keyboard elicited laughter and smiles. However, on return to the classroom, she became visibly anxious. Staff noted that she generally struggles to access the classroom due to her discomfort around group activities. When supported to post her photograph, she posted it independently on the ‘don’t like’ section, which was contraindicative to her perceived mood in the music therapy session. The familiar adult commented that there were three possible interpretations: (1) the young person was indicating that she did not enjoy the activity; (2) she did not feel comfortable the classroom; (3) she was simply matching an instant photograph of herself to her identifying photograph on the Talking Wall.

#### Theme 3: The Role of the Supporting Adult

One of the overriding themes that resurfaced at each stage of the project, and was discussed by staff in the evaluation interviews, was the role of the supporting adult. Although staff referred to the need for clarity as to ‘responsibility’ and ‘accountability’, it was generally concluded that the key issue is that all staff need to be able to interpret the young person’s communication bid. Staff felt that more training was required.

… [staff] want to go to the Wall, ask the child how they are feeling and them be able to use it straight away. And it’s getting it across to staff that actually it will take months, they won’t be able to use it to tell us how they are feeling yet – it’s about us using the Wall to model and I think that is going to be the difficulty, that it’s not a quick fix.

Another staff member concluded: “It’s really just about the knowledge – everyone having the knowledge.”

## Discussion

Existing research on methods to elicit voice in young people with autism and complex needs has been limited. The present study aimed to extend knowledge in this area by combining elements from a number of evidence-based methods, to create an innovative method that could be used inclusively for all young people at Prior’s Court School—a residential special school in the South East of England. Data collected from this exploratory research suggests that Talking Walls is a promising approach, worthy of further development.

Positive findings that stemmed from this research included how staff identified the need to work on supporting the expression of the emotions that underlie the young people’s preferences. Overall, staff reported that it was easier for the young people to evidence the positive aspects of life at the school, rather than the negative. These findings accord with research by Preece and Jordan (2010), who identified that children with autism experienced difficulties evaluating their emotions and using them to evaluate their daily life and social care support. Children in their sample could best identify preferences with regard to concrete topics such as food; however, identifying emotions regarding people, or reasons why they liked or disliked them, was more difficult. Staff at Prior’s Court identified this as a training need and, over the course of this project, became more confident to label and model their own negative emotions as ‘worried’ or ‘sad’. Recording this, however, remained difficult. In response, staff created an innovative solution: an envelope in which to ‘post’ negative experiences. This solution showed promise and is worthy of further development and evaluation.

The use of an adapted communication checklist to observe expressive language, understanding and social communication also showed promise. This enabled holistic observations, which highlighted that even when young people were able to express themselves verbally, they still relied on nonverbal or ‘pre-symbolic’ language to aid their communication bids. Standing close to their familiar adult, shifting eye gaze between the activity and the familiar adult, and varying facial expressions during an activity all provided valuable prompts as to how the young person was feeling. These prompts can be easily missed or can be difficult to interpret if the supporting adult is not familiar to the young person. This finding echoes the recommendations found in Hill et al. (2016), who suggested that these observations should form part of the wider education and therapeutic assessment cycle.

It is unsurprising that results confirmed issues arise around transition, as the pilot Talking Walls were in a fixed location. It is recognised that when young people with autism are required to change activity or location, these transitions can be stressful and may result in anxiety or behaviours that challenge (American Psychiatric Association 2013). The TEACCH approach (Mesibov et al. 2004), which was followed at Prior’s Court, encourages the use of visual schedules and/or transition objects to minimise the stress of transitions and provide the young people with the information about what they are doing, where and when. Previous research (Taylor and Preece 2010) has adapted some aspects of this approach to include objects, music and voice presented as transition symbols, and success was evidenced. In the same way, innovative solutions such as using an instant camera photograph aimed to keep the event ‘current’ and acted, in some way, as a transition ‘object’.

It is important to share not only the results of research projects, but also the problems and setbacks encountered during the process of the research (Beresford et al. 2004; Harrington et al. 2014; Scott-Barrett et al. 2019). This is especially important for the current study, since there is a general tendency to imply that the failure of research to demonstrate a ‘good’ outcome is due to a lack of fidelity by school-based practitioners (Howlin et al. 2007; Parsons et al. 2013; Stahmer et al. 2010) rather than limitations with the research. This project is still in the stages of defining and refining the Talking Wall and, as such, fidelity was not a critical concern at this stage. However, the resulting solutions often proposed by researchers were mirrored in this study, including improved implementation (e.g., more training for staff, with a particular focus on process: *how* to take young people to the Talking Wall and *how* to encourage peer interaction) and increased general awareness (e.g., wider information dissemination, accountability and responsibility), but this still implies that practitioners need to ‘fit in’ with what has been prescribed. Rather than seeking further solutions to improve outcomes, it is proposed, in this case, that more time is required to embed a new practice and effect change.

Regarding the limitations of the research, the study was fairly short, carried out over a six-month period, which was not long enough to fully embed the Talking Walls. Second, the study was carried out by an insider-researcher and, whilst this could be considered a benefit, it leads to a lack of researcher independence and leaves the study open to the accusation of bias (with the design of the project potentially guided by the intention of promoting a particular outcome favoured by the research setting; Robson 2002). However, as suggested by Parsons et al. (2013), the expertise and rigour of a partnering academic institution (and, in this instance, an independent academic researcher—the second author) goes some way to mitigate this factor. Third, although a range of employed ‘familiar adults’ participated in the project, the project did not extend to the voices of all key stakeholders, including parents. This would be an important avenue for future research, especially if a similar approach could be adopted in the family home. In addition, although the young people themselves were participants, they were observed and were therefore passive in their participation. As Milton et al. (2014) contend, this potentially adds to a knowledge base created by non-autistic others, rather than the ideal of fully participatory research.

Two further questions arose from the participation of the young people. First, because they are taught to use visuals to ‘match’ to schedules, it was difficult for staff to accurately assess if the young people were ‘matching’ symbols or displaying true communicative intent when using the Talking Wall. Second, there was the risk that young people were working for praise and thought that a positive response was the ‘correct’ response. This was further complicated by staff avoiding addressing negative emotions for fear that this could trigger behaviours that challenge. To mitigate this limitation, further studies should address the teaching of emotion in young people with autism and complex needs, both in terms of direct teaching for the young people and also further training for supporting adults. Studies involving family members would also be highly beneficial in this regard, comparing the behaviours and responses in the two settings of the family home and residential care.

Finally, the findings reported in this research are relevant to one group of young people, in one specific setting, and overgeneralisations should not be made. Yet this innovative approach does appear worthy of further development and it is hoped that this study goes some way to adding to the body of evidence evaluating innovative methods to hear the voices of young people with autism and complex needs.

## Electronic supplementary material

Below is the link to the electronic supplementary material.

Supplementary file1 (DOCX 20 kb)

## Footnotes

1 TEACCH (Treatment and Education of Autistic and Communication-Handicapped Children) promotes the use of structured teaching (which consists of four elements: physical structure; visual schedules; work systems; and task organisation) (Mesibov et al. 2004).

2 Focus group discussions highlighted the need to ‘link’ the education and residential settings of the school, and the suggestion was made that a scrapbook could be created to collect the items pinned from the Talking Wall. As the project was still at an exploratory stage, staff felt that more time was required to progress to this next development and, at the time of writing, only one pilot area of the school had trialled a scrapbook. This remains an area for future development.
