# Supplementary material for: The Development and Feasibility Study of a Multimodal ‘Talking Wall’ to Facilitate the Voice of Young People with Autism and Complex Needs: A Case Study in a Specialist Residential School
Source: J Autism Dev Disord. 2020 Apr 8;50(12):4267–79. doi: 10.1007/s10803-020-04476-6 (PMC7677153; doi:10.1007/s10803-020-04476-6)
Supplement: Supplementary file 1 — Supplementary file1 (DOCX 20 kb) [file 10803_2020_4476_MOESM1_ESM.docx]

**Supplementary material. How to use the Talking Walls: Instructions for Pupils and Staff**

**Script for social story produced in setting specific symbol software:**

**How to use the Talking Wall: Pupils**

Staff are here to help you

We want to know how you feel

You can use the talking wall to tell us

You can tell us what you like and don’t like

You can write.

You can stick.

You can draw.

You can take a photograph.

You can use the talking buttons.

This is ok

**How to use the Talking Wall: Staff**

The aim of the wall is to support our Young People to have a voice.

If we can support them to make their wishes and feelings known, we are increasing their independence and making sure that we are helping to place their best interests at the heart of decisions made about their lives.

To be able to express our wishes and feelings, we need to recognise and label our emotions. We know that this is challenging for our Young People, but we *can* support them.

1. **The wall**

Each environment identified in the pilot will have a wall space to gather likes/dislikes.

This space may look different in each area, but all walls should have the following parts

- Space for each Young Person
- A space for recording ‘I like’, ‘I don’t like’, ‘it’s ok’
- A social story to explain the purpose of the wall
- Paper 5x5 symbols of key emotions you can add and write comments on

The Young Person can post anything on the wall to represent something they like / don’t like. You can also support the Young Person to post items or model how to use the Talking Wall. This should be done during the activity or as soon after as possible as many Young People have difficulties reflecting on past events.

Things that could be put on the wall include:

- Photos of the Young Person during the activity
- Restaurant menu
- Cinema / theatre tickets
- Food wrappers
- Bowling score card
- Post-it notes that Young People can use to write or dictate their opinion to staff
- Leaflet from a day trip

Blue tack can be used to place the items on the wall, so they can easily be taken off on a weekly basis to record in their scrapbook.

Although it is important to record things that the Young Person doesn’t like, they may find it distressing to see these things on the wall. One idea is that the ‘don’t like’ section could have an envelope where you put the ‘don’t like’ items so that these are still recorded, but out of sight.

1. **Modelling emotional language**

Many of our Young People struggle understanding and expressing ‘like’ ‘don’t like’ and being able to appropriately say when they feel ‘happy’, ‘sad’, ‘angry’ or ‘worried.’ To help with this, we need to be using key emotion words throughout the day for the Young Person to hear. Our language can still be minimal, but we can also alter our tone of voice as we are commenting on how we feel.

So, which emotions should we focus on? A good place to start is with basic emotions that have a clear physical response:

- **Happy**
- **Worried**
- **Sad**

Of course, we don’t always need to express a feeling about something, or maybe we just don’t know *how* we feel. So, another useful option is:

- **I don’t know**

1. **Labelling the young person’s emotions**

The best way to teach a feeling word is to say the word when you think the young person is feeling it… that way they are hearing the word when they are feeling the sensation from the emotion.

The wall is a space for the young person to visualise their feelings. Let’s think about some examples of how this could work:

1. During an art lesson, the Young Person appears to enjoy painting an Easter egg. You could say “**You are smiling, I think you are happy**.” Repeat this phrase as the Young Person smiles or shows other body language that you know to be a signal that they are having a positive experience. If it is possible to take the finished work and support the Young Person to stick it onto the wall in the ‘like section’ (or do it on their behalf), this reinforces the emotion if you repeat again, “**I think you are happy**”. You could add a happy visual symbol to this work to reinforce the emotion further.
2. A trip to the supermarket involved an experience with a dog, tied up outside the shop. The Young Person has a fear of dogs, but with support, handled the situation well. During the experience, you could say to the young person “**you are pulling away from me. I think you are worried. It is ok to be worried, but you are safe.**” It is important to make room for negative emotions and not ‘shut them down’ by saying “Don’t be worried. Stop that now.” However, if the experience happens away from the school or home environment, it is difficult to use the wall to reinforce this experience immediately and because it is negative, it would not be necessary for the Young Person to relive that experience later. However, you, as the supporting adult, could write an observation note when you are back at school / in house and post it on the Young Person’s behalf.
3. The score card from a trip to the bowling alley could represent a ‘happy’ experience. Whilst at the bowling alley you could say “**you are jumping up and shouting ‘strike!’, I think you are happy**”. You could take a photograph of their smiling face and show them the image on the camera, repeating “**you are smiling, I think you are happy**”. Take the score card home and print out the photograph. Support the Young Person to post them both on the wall saying “**you shouted strike! I think you liked bowling**” Ask them to place the card on the wall in the like section and add a happy symbol to it.
4. During a trip to a restaurant, the Young Person does not appear to enjoy their usual favourite meal. You could take a photograph and say, “**I can see that you have left the fish, I don’t think you like it.**” On return to house, you could ask the Young Person to post the photograph on the wall. Make a note of where they post the picture compared to your observation.
5. A Young Person enjoys selecting music to listen to that week and you decide to use the wall to help them decide which songs will be played, you encourage them to sort the songs into ‘like and don’t like’. Your language to support them could include “**I can see you don’t like ‘Humpty Dumpty’. I think you feel sad/worried when you sing/hear Humpty Dumpty**.” Also, you could record them singing their preferred songs on a talking tile and that could be stuck on the wall under the ‘like’ area, to allow that Young Person to replay and listen to their happy voice. You could support this with “**I can hear you singing ‘Twinkle little star’ and I think you are happy”** and a written note.
6. **Creating a record of the Young Person’s wishes and feelings**

So, we have created a wall to allow for the space and the opportunity for the Young People to express their wishes and feelings… but we need to act on those wishes and feelings to show that we value our Young People and that we are listening and responding.

1. **Weekly Scrapbook**

By taking the evidence from the wall on a weekly basis and setting up an activity for the Young Person to stick this into a scrapbook we can maintain the wall as an interactive space.

A weekly scrapbook session can be timetabled into their waking day. This also provides another opportunity to talk about the week’s experiences. The Young Person should have the choice as to what they include in the scrapbook. Questions like “would you like to visit… again?” reinforce the concept that the Young Person has a voice in determining their schedule. Any opinion that the Young Person voices can be positively acted upon and you can write notes in the book to record the choices they make

1. **Evidence**

Teachers and Home Managers can monitor the scrap books and use as evidence.

1. **Useful phrases**

On the wall, you will find emotion symbols.

Phrases to help you describe emotions:

- I think that you feel…
- I can see that you are… right now.
- You look…
- You seem…

Remember: your body language, facial expression and tone of voice also have an effect.
